# Dermatoscopy of Neoplastic Skin Lesions: Recent Advances, Updates, and Revisions

**DOI:** 10.1007/s11864-018-0573-6

**Published:** 2018-09-20

**Authors:** Philipp Weber, Philipp Tschandl, Christoph Sinz, Harald Kittler

**Affiliations:** Department of Dermatology, Medical University of Vienna, Währinger Gürtel 18-20, 1090 Vienna, Austria

**Keywords:** Dermoscopy, Dermatoscopy, Melanoma, Basal cell carcinoma, Squamous cell carcinoma, Actinic keratosis

## Abstract

Dermatoscopy (dermoscopy) improves the diagnosis of benign and malignant cutaneous neoplasms in comparison with examination with the unaided eye and should be used routinely for all pigmented and non-pigmented cutaneous neoplasms. It is especially useful for the early stage of melanoma when melanoma-specific criteria are invisible to the unaided eye. Preselection by the unaided eye is therefore not recommended. The increased availability of polarized dermatoscopes, and the extended use of dermatoscopy in non-pigmented lesions led to the discovery of new criteria, and we recommend that lesions should be examined with polarized and non-polarized dermatoscopy. The “chaos and clues algorithm” is a good starting point for beginners because it is easy to use, accurate, and it works for all types of pigmented lesions not only for those melanocytic. Physicians, who use dermatoscopy routinely, should be aware of new clues for acral melanomas, nail matrix melanomas, melanoma in situ, and nodular melanoma. Dermatoscopy should also be used to distinguish between different subtypes of basal cell carcinoma and to discriminate highly from poorly differentiated squamous cell carcinomas to optimize therapy and management of non-melanoma skin cancer. One of the most exciting areas of research is the use of dermatoscopic images for machine learning and automated diagnosis. Convolutional neural networks trained with dermatoscopic images are able to diagnose pigmented lesions with the same accuracy as human experts. We humans should not be afraid of this new and exciting development because it will most likely lead to a peaceful and fruitful coexistence of human experts and decision support systems.

## Introduction

Dermatoscopy (dermoscopy) is a non-invasive diagnostic method for the examination of pigmented and non-pigmented skin lesions [1–3, 4••]. It improves the early detection of melanoma in comparison with inspection with the unaided eye and impacts therapy and management [5–8]. Although other non-invasive diagnostic techniques such as in vivo reflectance confocal microscopy and optical coherence tomography have a better resolution, dermatoscopy is still the state-of-the-art method for evaluating pigmented and non-pigmented skin lesions because it is accurate, fast, easy to use, and widely available [4••, 9]. Meta analyses showed that, in comparison with the unaided eye, dermatoscopy increases the diagnostic sensitivity for melanoma up to 25% depending on the experience of the physician [10–13]. In recent years, studies focused on the revision of melanoma criteria, on dermatoscopic criteria of melanoma on special sites and of specific melanoma subtypes, on non-pigmented neoplasms, and, last but not least, on machine learning to train computer algorithms to diagnose dermatoscopic images without the need of human expertise.

## New algorithms and revision of criteria

The most widely used algorithms to differentiate between melanomas and nevi by dermatoscopy are the ABCD rule of dermoscopy, Menzies method, the 7-point checklist, and the chaos and clues algorithm. The chaos and clues algorithm is relatively new and was introduced by Rosendahl et al. [14]. In essence, it is a condensed variant of pattern analysis. In its original version, it defined eight melanoma clues: eccentric structureless zones of any color (except skin color), gray or blue structures, black dots or clods in the periphery, segmental radial lines or pseudopods at the periphery, white lines, thick reticular lines, polymorphous vessels, and parallel lines on the ridges (for acral lesions). The clues are similar to the clues used in the other algorithms.

The advantage of the chaos and clues algorithm is that it uses chaos (asymmetry of color or structure) as a screening tool and, because it does not need complicated calculations, is quick and easy to use. Unlike other algorithms, it can be used for all pigmented lesions not only for those melanocytic. The clues of the algorithm work not only for melanoma but also for pigmented basal cell carcinoma (pBCC) and pigmented squamous cell carcinoma (pSCC).

**Fig. 1:**
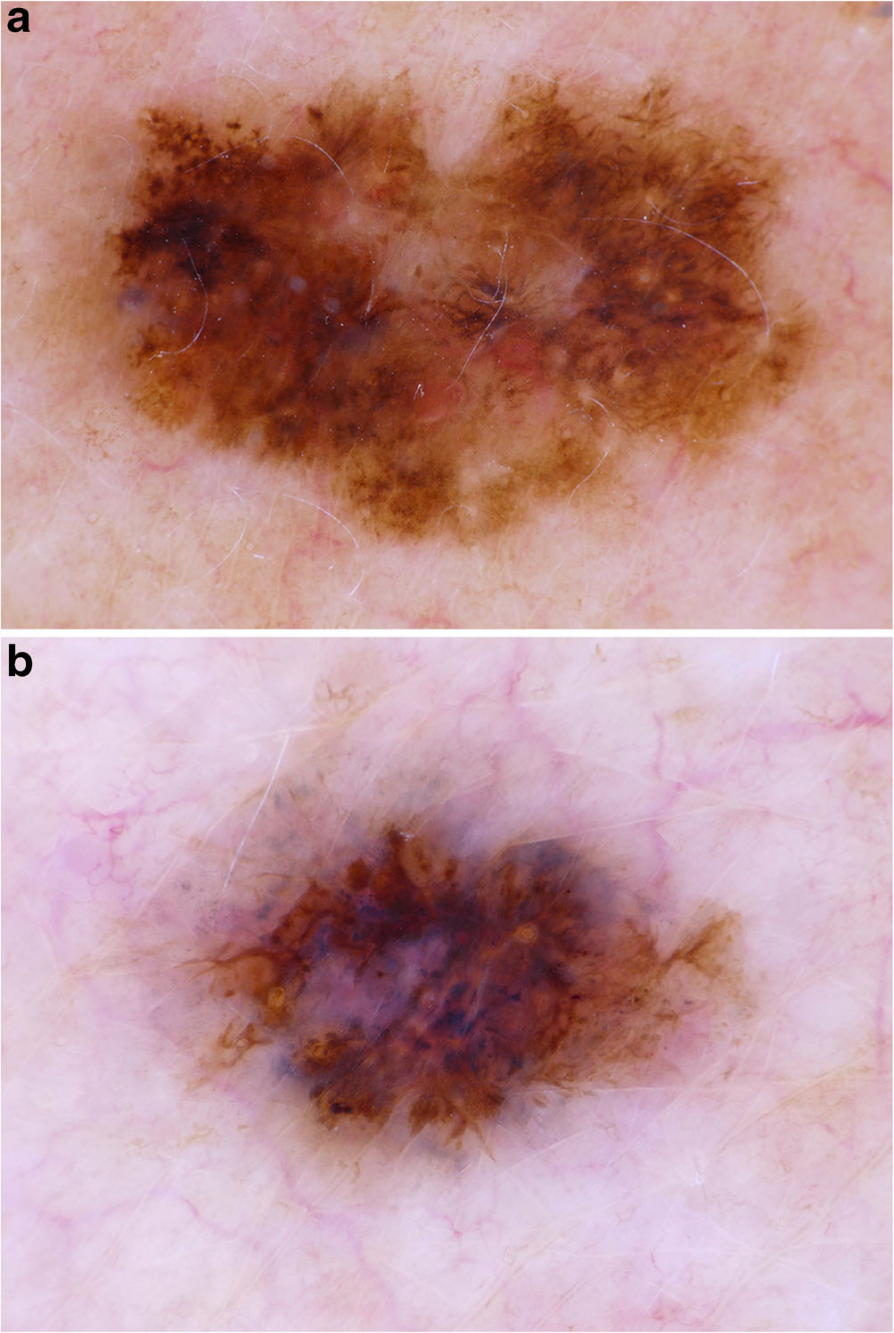
Chaos and clues in melanoma. **a** Asymmetry of structure and color (“chaos”); clues to malignancy: eccentric structureless zone, gray dots, on the left side, black dots in the periphery, thick reticular lines. Histopathologic diagnosis: melanoma. **b** Asymmetry of structure and color (“chaos”); clues to malignancy: eccentric gray and blue structureless zone, black dots in the periphery, segmental radial lines at the periphery, white lines. Histopathologic diagnosis: melanoma. Images courtesy of the Vienna Dermatologic Imaging Research Group, Department of Dermatology, Medical University of Vienna, Austria.

In short, the examiner has to apply two steps: the first step is to evaluate the pigmented lesion dermatoscopically for “chaos,” defined as “asymmetry of structure or colour.” If chaos is present, the second step is to search for the eight clues to malignancy mentioned above. If one or more clues are found, a biopsy is performed unless an unequivocal diagnosis of seborrheic keratosis can be made by pattern analysis. If no chaos is present, the examiner moves to the next lesion **(**Fig. 1**)**.

Jaimes and coworkers [15] described new criteria for melanomas on chronic sun-damaged skin. One of these criteria, angulated lines, was added to the chaos and clues algorithm because it is a specific feature of flat melanomas on chronic sun-damaged skin.

The importance of chaos was also confirmed by others. In 2017, Carrera and coworkers [16] revisited the dermatoscopic criteria of melanoma and demonstrated that features that quantify the overall architecture of a lesion (architectural disorder, contour asymmetry, pattern asymmetry) have high levels of interobserver agreement and discriminatory power. Local features on the other hand, especially those with low prevalence like “atypical network” or “irregular blotch” had a poor agreement and low discriminatory power.

### In situ melanoma

**Fig. 2:**
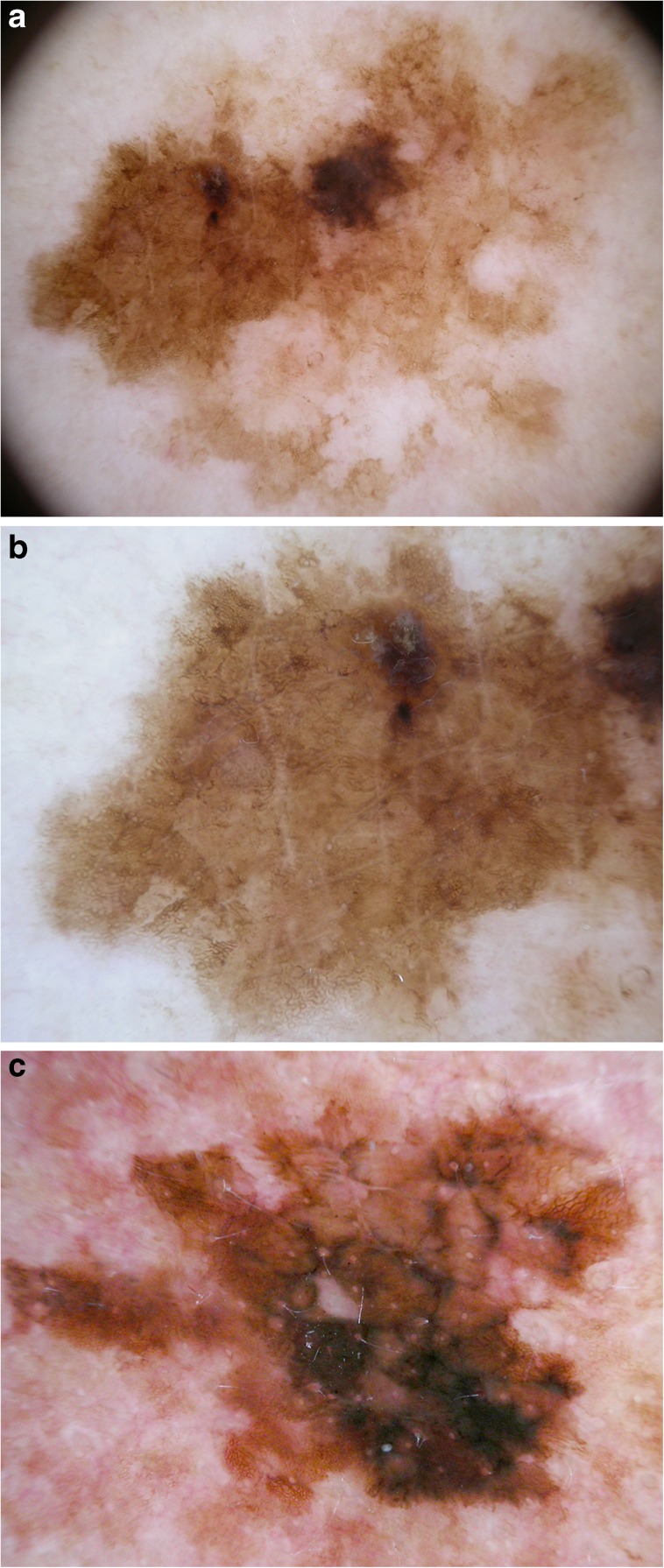
Prominent skin markings and angulated lines in melanoma in situ. **a** Dermatoscopic image of a melanoma in situ on the thigh of a 68-year-old man. **b** In detail, lesion (**a**) shows prominent skin markings (linear intersecting furrows) as a clue to melanoma in situ. **c** Angulated lines and eccentric irregular hyperpigmented area as clues to melanoma in situ. Images courtesy of the Vienna Dermatologic Imaging Research Group, Department of Dermatology, Medical University of Vienna, Austria.

Ramji et al. recently demonstrated that melanomas in situ usually are chaotic and that most in situ melanomas show at least two clues to malignancy [17]. In another study, Lallas and coworkers compared the dermatoscopic criteria of melanoma in situ with benign lesions that commonly mimic melanoma in situ [18••]. They identified five variables (atypical network, regression > 50%, irregular hyperpigmented areas, angulated lines, and prominent skin markings) as significant clues to melanoma in situ **(**Fig. 2**)**. In a direct comparison of melanoma in situ with atypical (“dysplastic”) nevi, the presence of irregular hyperpigmented areas and the presence of prominent skin markings proved to be particularly useful clues to differentiate melanoma in situ from atypical nevi.

### Nodular melanoma

The nodular subtype accounts for 10–30% of all melanomas but comprises a high percentage (37%) of fatal cases [19, 20••]. Nearly 50% of all melanomas thicker than 2 mm are nodular melanomas. The “ABCD” warning signs work better for superficial spreading melanomas than for nodular melanoma. For nodular melanoma, Kelly [21] introduced the “EFG” rule (elevation, firmness on palpation, continuous growth over 1 month). According to Menzies et al. [22], nodular melanomas are more frequently amelanotic or hypomelanotic (37.3%) than other melanoma subtypes (7.5%). If nodular melanomas are pigmented, they are typified by structureless blue pigmentation, predominant peripheral vessels, and areas of pink and black colors. In the study by Menzies et al., a small percentage of nodular melanomas (5.8%) was not chaotic with regard to structure or color. It is important to know that nodular melanomas may simulate dermal nevi, Spitz nevi, dermatofibromas, and vascular lesions. In a more recent study, Pizzichetta et al. [20••] reported that nodular melanomas are typified by ulceration, blue structureless areas (“blue veil”), multiple colors and asymmetric pigmentation, and the combination of polymorphous vessels and milky-red clods (globules).

### Acral melanoma

In non-white populations, acral melanoma represents the most common subtype accounting for > 70% of melanomas in African Americans and 50% of melanomas in Asian patients. It accounts for less than 10% of cases in white patients [23–27]. The classic dermatoscopic criteria of acral melanoma have been described in an Asian population by Saida and coworkers [28]. They found that the parallel ridge pattern is the most specific clue for flat acral melanomas on dermatoscopy.

Braun et al. [29] analyzed 66 acral melanomas in a predominantly Caucasian population. In this retrospective study, the authors differentiated between easy, moderate, and very challenging lesion depending on diagnostic difficulty. The parallel ridge pattern, chaos (termed “bizarre pattern” by Braun et al.), milky-red areas, and structureless pigmentation (termed “diffuse pigmentation” by Braun et al.) were the most important dermatoscopic criteria for acral melanoma.

A recent study [30••] that included 131 acral melanomas confirmed the importance of the parallel ridge pattern, which reached a 99% specificity for melanoma. On the other hand, it was present only in 38% of acral melanomas. The moderate sensitivity of the parallel ridge pattern indicates the need for additional criteria for acral melanoma.

**Fig. 3:**
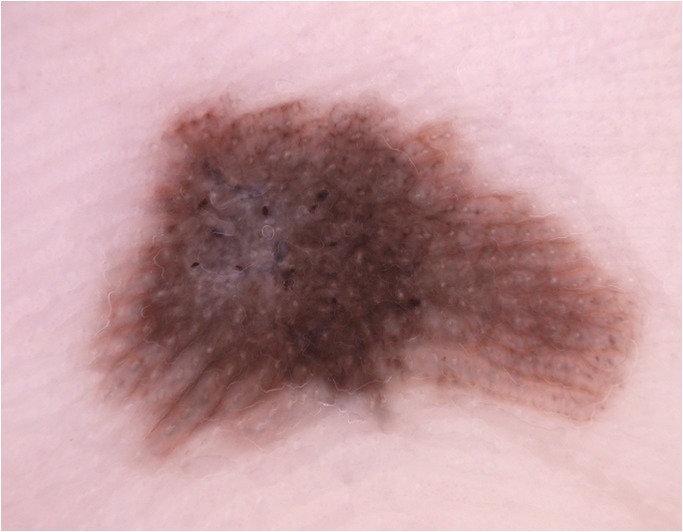
Acral melanoma. Dermatoscopic image of an acral melanoma with asymmetry of structures (+ 1 point), asymmetry of color (+ 1 point), irregular blotch (+ 1 point), and the parallel furrows pattern (− 1 point) resulting in a BRAAFF-Score of 2 points. Despite absence of the parallel ridge pattern, it can be diagnosed as melanoma. Image courtesy of the Vienna Dermatologic Imaging Research Group, Department of Dermatology, Medical University of Vienna, Austria.

Lallas et al. [30••] proposed a score **(**Table 1**)** with four positive (irregular blotches, asymmetry of colors, asymmetry of structure, and the parallel ridge pattern) and two negative features (parallel furrow pattern and fibrillar pattern) **(**Fig. 3**)**.

**Table 1:** The *BRAAF Checklist* for the diagnosis of acral melanoma [30••]

Acronym	Criterion	Points
B	Irregular blotch	+ 1
R	Parallel ridge pattern	+ 3
A	Asymmetry of structures	+ 1
A	Asymmetry of colors	+ 1
F	Parallel furrow pattern	− 1
F	Fibrillar pattern	− 1

A total score of ≥ 1 is needed for a diagnosis of melanoma

The chaos and clues algorithm described earlier can also be used for acral lesions because irregular blotches, which correspond to eccentric structureless areas, asymmetry of color, and asymmetry of structure can also be summarized under the term chaos.

The score for acral lesions developed by Lallas achieved a sensitivity of 93.1% and a specificity of 86.7%. In addition, the authors proposed four simple management rules for acral lesions: symmetric lesions with a parallel furrow or a fibrillar pattern (crossing pattern) are most probably benign and should not be excised. Lesions with a parallel furrow or fibrillar pattern should be excised if there is asymmetry (chaos). Lesion lacking lines (ridge, furrow, or fibrillar pattern) should be excised in the presence of asymmetry or in the presence of irregular blotches.

### Facial melanoma

The classic dermatoscopic criteria of facial melanoma in situ (lentigo maligna) have been described by Stolz and coworkers [31]. The differential diagnosis of flat pigmented facial lesions is challenging because pigmented actinic keratosis (pAK), lichen planus-like keratosis (a solar lentigo with lichenoid inflammation and regression), and lentigo maligna may look very similar dermatoscopically.

In an attempt to better characterize the dermatoscopic patterns of flat pigmented facial lesions, Tschandl et al. [32] used a pattern analysis to differentiate between the various types of lesions. They found that certain patterns are more specific than others. A pattern of circles for example is relatively specific for early lentigo maligna, especially if the circles are thin and gray. Solar lentigines on the other hand are typified by brown pigmentation only and a reticular pattern, a structureless pattern, or a pattern of curved lines (“fingerprint pattern”). Pigmented actinic keratosis (pAK), however, have no specific pattern and may mimic lentigo maligna. Another difficulty is that many cases of pAK are collisions of an actinic keratosis with a solar lentigo, which explains why parts of pAK have patterns typical of solar lentigo. Tschandl et al. were able to demonstrate that the best clues to pAK were white circles, scale, and rosettes (four dots arranged in square), a feature that is better seen with polarized dermatoscopy. This also confirmed the work of Akay et al. who were the first to describe the dermatoscopic criteria of pAK in detail [33]. Another important finding of the study by Tschandl et al. was the confirmation that the color gray, i.e., the presence of gray structures, is an important clue to malignancy in facial lesions. The presence of gray had a high sensitivity (85.1%) but a low specificity (39.7%).

**Fig. 4:**
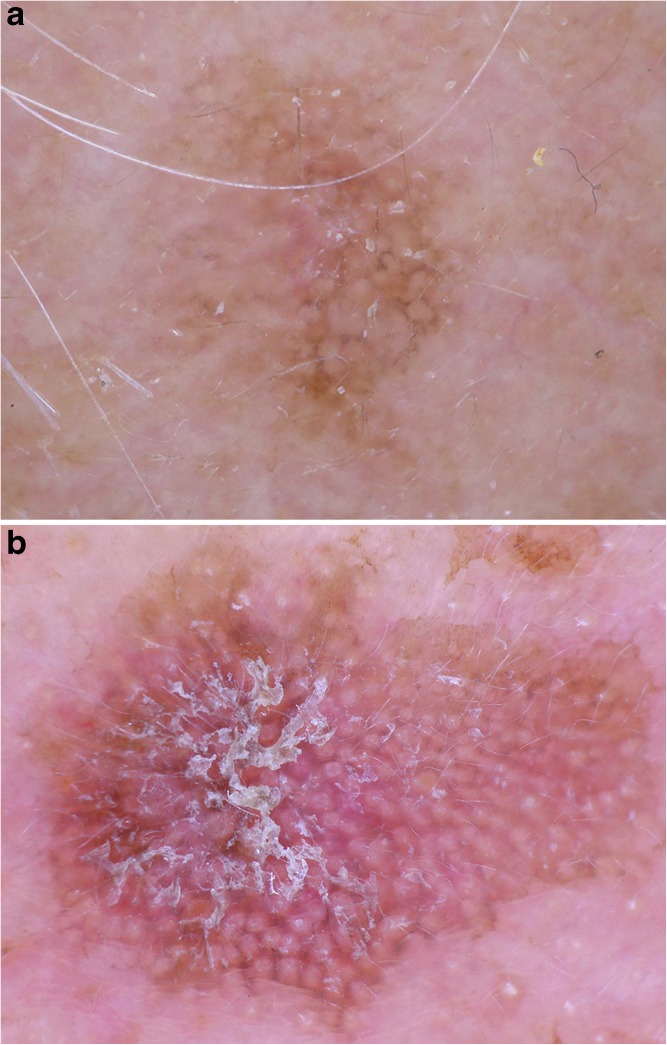
Pigmented actinic keratosis. Lesions (**a**) and (**b**) show surface scaling and follicular openings. Additionally, characteristic rosettes (4 dots arranged in square) can dermatoscopically be seen in (**b**). The presence of angulated lines in both lesions are in line with the diagnosis of pigmented actinic keratosis. Images courtesy of the Vienna Dermatologic Imaging Research Group, Department of Dermatology, Medical University of Vienna, Austria.

Lallas et al. [34] compared the dermatoscopic appearance of lentigo maligna and pAK. They found that gray rhomboids (angulated lines), non-evident follicles, and intense pigmentation were associated with lentigo maligna. They confirmed that white circles were the most specific clue for pAK. Like other studies they also confirmed the importance of scale and red color for the diagnosis of pAK **(**Fig. 4**)**.

In 2017, Tschandl et al. [35••] published a new approach to differentiate lentigo maligna from benign facial lesions. Instead of focusing on the criteria of lentigo maligna, they choose to focus on the dermatoscopic criteria of benign lesions. They recommend to look for features of benign lesions first. These features are scales, white follicular openings, erythema or reticular vessels, reticular or curved lines (fingerprints), structureless brown color, sharp demarcation, and classic criteria of seborrheic keratosis. If none of these features are present lentigo maligna should be considered.

### Nail pigmentations and melanoma of the nail matrix

Longitudinal melanonychia is characterized by brown or black pigmented lines extending from the proximal nail fold to the distal end of the nail plate. Dermatoscopy can distinguish between nevi and melanoma of the nail matrix. Thomas et al. [36] described the classic criteria of nail matrix melanoma and published clues to differentiate melanoma from nevi and also from other benign causes of longitudinal melanonychia such as lentigo of the nail matrix, ethnic type hyperpigmentation, trauma-induced hyperpigmentation, drug-induced hyperpigmentation, and subungual hemorrhage. The decision to biopsy the nail matrix is difficult because it is a painful procedure and can cause permanent nail dystrophy. Information beyond dermatoscopy such as age, history, and involvement of other nails play an important role and should be included in the decision process. Because congenital nevi of the nail matrix may mimic melanoma and nail matrix melanoma in prepubescent children is exceedingly rare, a nail matrix biopsy in this age group should be considered only in exceptional cases.

**Fig. 5:**
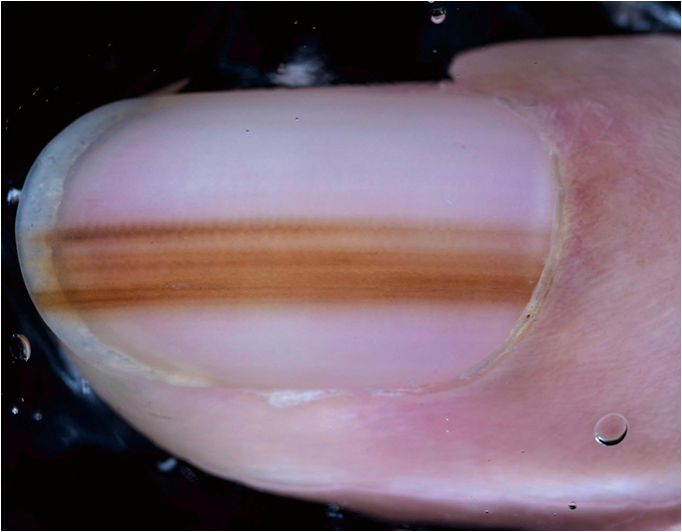
Acral lentiginous melanoma in situ of a 37-year-old woman. Dermatoscopy shows longitudinal lines of irregular thickness, spacing, and coloration. Note that the pigmentation involves less than $$ \raisebox{1ex}{$1$}\!\left/ \!\raisebox{-1ex}{$3$}\right. $$ of the nail plate, demonstrating that size is not a good criterion for early lesions. Image courtesy of the Vienna Dermatologic Imaging Research Group, Department of Dermatology, Medical University of Vienna, Austria.

The International Dermoscopy Society (IDS) reevaluated the dermatoscopic criteria of longitudinal nail pigmentations and tried to identify the best predictors of nail melanoma [37••]. The retrospective study included 25 nail apparatus melanomas and 57 benign lesions (32 melanocytic nevi and 25 benign melanocytic hyperplasia). They confirmed that melanoma of the nail apparatus involves the first digit more frequently than other digits. In melanomas the pigmentation typically involved more than 2/3 of the nail plate whereas most benign lesions showed less than 1/3 of nail plate involvement. Other important clues to melanoma were gray and black color, irregularly pigmented lines, the Hutchinson- and the micro-Hutchinson signs, and nail dystrophy such as thinning, splitting, or partial or total absence of the nail plate **(**Fig. 5**)**. If nail dystrophy was present, the odds for melanoma were three times higher.

Ohn and coworkers recently presented a scoring model for subungual melanoma in situ based on a logistic regression of established dermatoscopic criteria [38]. In this score asymmetry, the Hutchinson sign, and a width of pigmentation of at least 6 mm count 2 points, and border fading, multicolor pigmentation, and a width of pigmentation of at least 3 mm count 1 point. The total score ranges from 0 to 8 points. The authors suggest a cutoff value of 3 points to distinguish benign melanonychia from melanoma in situ. At this cutoff, the score had a sensitivity of 89% and a specificity of 62%.

In another study, Ohn et al. investigated dermatoscopic characteristics of nail matrix nevus in children and compared them with nevi of adults [39]. Nevi in children were darker and more often multicolored than nevi of adults. Melanoma clues like chaos (“irregular pattern”), the Hutchinson sign, the pseudo-Hutchinson sign, and the triangular sign were more frequent in nevi of children.

## Dermatoscopy of non-melanoma skin cancer (keratinocyte cancer)

The term “non-melanoma skin cancer” is a generic term that has been used to combine basal-cell carcinomas (BCC) and squamous-cell carcinomas (SCC). Recently, Karimkhani et al. [40] suggested that the term “non-melanoma skin cancer” should be replaced by “keratinocyte cancer.” We think that generic terms like “non-melanoma skin cancer” and “keratinocyte cancer” are expendable, and BCC and SCC should be addressed specifically whenever possible.

**Fig. 6:**
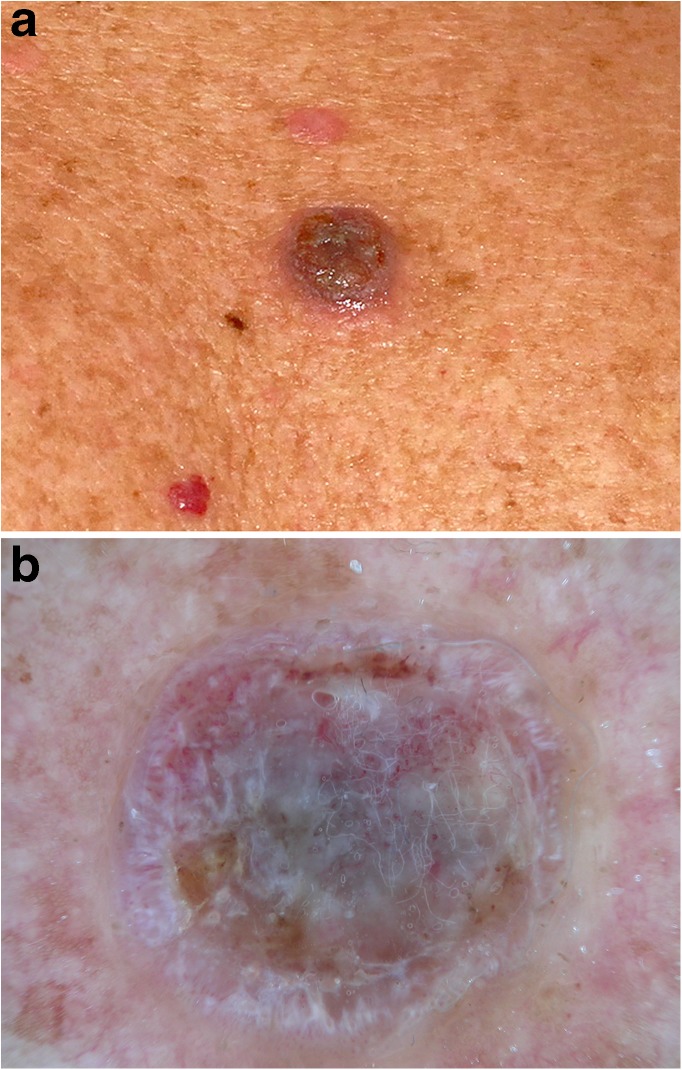
Hypopigmented nodule on the back of a 72-year-old man viewed with the unaided eye. **a** Dermatoscopic examination of lesion (**b**) shows ulceration with serum crusts and adherent fibers. Central gray color surrounded by white lines and coiled vessels allows the differential diagnosis of an amelanotic melanoma. Histopathologic diagnosis: nodular melanoma (Clark level, IV; Breslow depth, 2.4 mm; ulceration, stage T3b). Images courtesy of the Vienna Dermatologic Imaging Research Group, Department of Dermatology, Medical University of Vienna, Austria.

BCCs and SCCs belong to the most common human cancers with a rising incidence [41–46]. Whether actinic keratoses and Bowen’s disease (intraepithelial carcinoma) should be regarded as in situ variants of SCC or as precursors is still a matter of debate. Although pigmented variants exist, both neoplasms are usually non-pigmented. Because dermatoscopic criteria are less specific and the number of differential diagnoses is significantly higher, non-pigmented neoplasms are more difficult to diagnose than pigmented lesions. In a recent study, however, Sinz et al. [4••] were able to demonstrate that dermatoscopy also improves the accuracy of non-pigmented lesions in comparison with inspection with the unaided eye, although the improvement was less pronounced than for pigmented lesions. The proportion of correct diagnosis of expert readers increased from 41.3% with the unaided eye to 52.7% with dermatoscopy. The improvement of non-experts was less pronounced. Dermatoscopy also improved the ability of expert readers to select appropriate management decisions **(**Fig. 6**)**.

### Basal cell carcinoma

Lallas and coworkers revised the dermatoscopic criteria of BCC and added some new observations [47]. They confirmed the importance of the classic BCC criteria such as branched (arborizing) vessels, blue clods (blue ovoid nests), blue or gray dots and globules, peripheral radial lines (maple leaf like areas), radial lines converging at a central dot (spoke-wheel areas), and ulceration or small erosions. They also discussed new criteria such as concentric structures (clod in a clod) and shiny white structures.

Navarette et al. [48••] evaluated the diagnostic significance of different types of shiny white structures, i.e., blotches, strands, and short white lines. Although shiny white structures can be found in a variety of malignant neoplasms including BCC, SCC, and melanoma, they found that the coexistence of shiny white blotches and strands is typical for BCC. Navarette et al. suggested that shiny white structures should be added to the BCC criteria. The classic BCC criteria described by Menzies [49] did not include shiny white structures because they are better seen with polarized dermatoscopy than with traditional contact fluid dermatoscopy and handheld polarized dermatoscopes did not exist when Menzies et al. published their work.

Some studies [50–52] also tried to differentiate between superficial basal cell carcinomas and other subtypes by dermatoscopy, which may have important consequences for treatment. According to Lallas et al. the presence of maple leaf-like areas (peripheral radial lines) with superficial fine telangiectasias is typical for superficial BCC. Additional dermatoscopic features of superficial BCCs are multiple small erosions and white or red structureless areas [50].

Superficial BCCs usually lack blue ovoid nests (blue clods) and arborizing (branched) vessels.

**Fig. 7:**
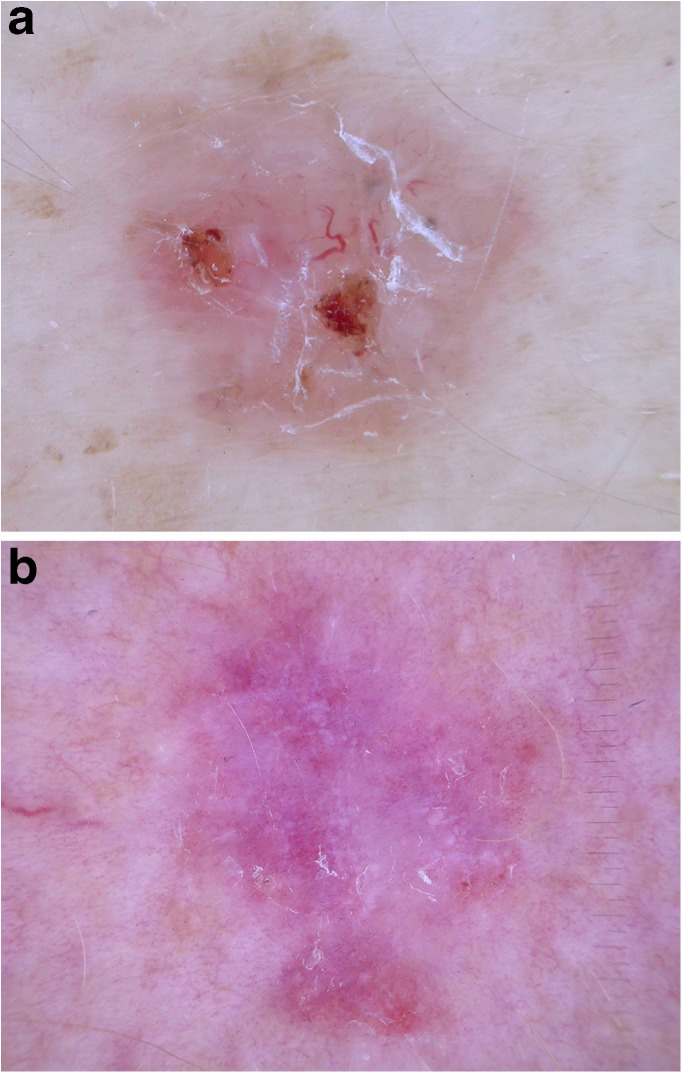
Lesion (**a**) shows the typical features of an infiltrative BCC such as branched vessels and small blue clods. The superficial BCC in (**b**) is typified by white lines. Images courtesy of the Vienna Dermatologic Imaging Research Group, Department of Dermatology, Medical University of Vienna, Austria.

Nodular BCCs on the other hand are typified by arborizing (branched) vessels and a single ulceration. Large blue ovoid nests (blue clods), blue dots and globules, and arborizing (branched) vessels are characteristic dermatoscopic findings of pigmented nodular BCC **(**Fig. 7**)**.

According to Zalaudek et al., the arborizing vessels of sclerodermiform BCCs are more scattered and thinner and show fewer branches compared with the arborizing vessels of nodular BCC [53]. White and red structureless zones are another typical feature of infiltrative or sclerodermiform BCC. Another typical sign for infiltrating growth of BCC is a so-called “stellate pattern.” This term was used to describe radial vessels and radial white lines in the periphery of the lesion [54].

Basosquamous carcinoma is a complex neoplasm characterized by basaloid and squamoid differentiation [55, 56]. The dual differentiation is reflected by the coexistence of dermatoscopic features of basal cell carcinoma and squamous cell carcinoma. According to Akay et al., basosquamous carcinoma is typified by a combination of BCC-dominant vascular features such as branching (arborizing) vessels and SCC-dominant morphology such as signs of keratinization [57].

### Squamous cell carcinoma and keratoacanthoma

Cutaneous SCC is the second most common skin cancer, with a rising incidence over recent decades. The most frequent sites of SCC are the head and neck area. Invasive SCC usually is elevated and, depending on differentiation, hyperkeratotic or ulcerated. Keratoacanthoma can be regarded as a highly differentiated variant of SCC [45, 58] although some authors regard it as a benign neoplasm [59].

Rosendahl et al. [60] demonstrated that the significance of clues depends on the context and the differential diagnosis. They compared dermatoscopic criteria of highly differentiated invasive SCC including keratoacanthomas with BCC, actinic keratosis, and Bowen’s disease. They found that coiled vessels are a strong clue to SCC when compared with BCC but are not helpful when the differential diagnosis is actinic keratosis and Bowen’s disease. In contrast, white circles, blood spots, and white structureless zones are typical for highly differentiated SCC when compared with actinic keratosis and Bowen’s disease. The strongest clue, regardless of the differential diagnosis, is the presence of keratin, especially in conjunction with blood spots. If the keratin plug is in the center of the lesion, the histopathologic diagnosis is more likely keratoacanthoma and not SCC.

As already mentioned, SCC can be highly or poorly differentiated, which is important for treatment and follow-up. Lallas et al. [61••] tried to specify dermatoscopic criteria that allow to differentiate between highly and poorly differentiated SCCs in vivo.

**Fig. 8:**
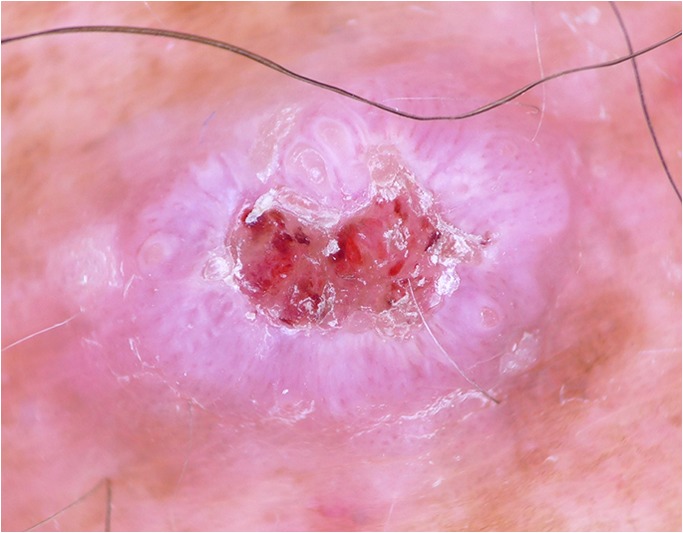
Highly differentiated SCC, in a 72-year-old man. Dermoscopy shows keratin with blood spots in the center and white circles in the periphery. Image courtesy of the Vienna Dermatologic Imaging Research Group, Department of Dermatology, Medical University of Vienna, Austria.

Poorly differentiated SCCs showed predominantly red color, which can be attributed to the absence of scaling and keratin and the presence of bleeding and ulcerations or dense vascularity. The quantity and the caliber of vessels significantly correlated with the grade of differentiation. If vessels covered more than 50% of the lesion and small-caliber vessels dominated then the lesion was more likely a poorly differentiated SCC. The presence of keratin, white circles, and white structureless was in favor of moderately or highly differentiated SCCs **(**Fig. 8**)**.

## Automated diagnosis of dermatoscopic images

Digital dermatoscopic images of skin lesions are easily available for automated diagnosis and machine learning. Binder et al. demonstrated already in 1994 that artificial neural networks can be trained successfully to distinguish melanoma from nevi using only dermatoscopic images as input [62, 63]. Although automated system works well in experimental settings and showed expert-level performance in systematic reviews [64], there is currently no widespread clinical application of such automated systems. This discrepancy can be partly explained by the lack of large, randomized, prospective clinical trials, and by the potential importance of information beyond image data such as history, age, and anatomic site.

More recently, convolutional neural networks (CNN) gained attention as machine-learning models for image classification because they outperformed older models by far [65]. Other advantages of CNNs are that segmentation of the lesion from the background and definition of features in advance are not mandatory. A disadvantage of CNNs is that they need a high number of images if they are trained from scratch. With a technique called “transfer learning ” which reuses features extracted from other image sources, it is, however, possible to train CNNs with a significant lower number of dermatoscopic images and still reach a high accuracy. With the use of transfer learning, CNNs trained with dermatoscopic images were able to reach an accuracy for melanoma on par or superseding humans [66] and board-certified dermatologists [67].

It is still unclear if experts will accept a decision support system and how it can be implemented in the daily workflow. Even a highly accurate automated system does not guarantee successful implementation in practice, and unforeseen problems can arise during application. In a prospective trial that compared the diagnostic accuracy for melanoma of dermatologists with and without decision support of an automated diagnostic system, melanomas were missed in the decision support arm simply because they were not selected for imaging by the physician [68]. Another problem is that the research on automated diagnosis of dermatoscopic images focused on the dichotomous decision of nevus versus melanoma [67] and largely ignored non-melanocytic lesions, which represent a significant portion of pigmented lesions selected for biopsy or excision [69]. Accurate predictions of multiple disease classes [66] may be more helpful in clinical practice but public image databases for this problem were not available until recently. The “ISIC 2018: Skin Lesion Analysis Towards Melanoma Detection” challenge was the first use of the HAM10000 dataset [70••], which is a public dataset of more than 10,000 dermatoscopic images of seven disease categories (melanoma, nevi, basal cell carcinoma, seborrheic keratosis/solar lentigo, dematofibroma, vascular lesions, and pigmented actinic keratoses/Bowen’s disease). The challenge attracted more than 100 participants and the winner algorithm reached an average sensitivity of 88.5% across all disease categories. The machine learning algorithms that participated in the challenge will be compared with human experts in a large reader study to show whether they can demonstrate expert-level performance for multiple disease categories.

## Summary

Dermatoscopy is still an active field of research. Dermatoscopic criteria for neoplastic skin lesions are constantly revised, added, or reinterpreted. The chaos and clues algorithm, which provides a reasonable diagnostic accuracy for malignant pigmented lesion while being considerably easy to learn and apply, is a good start for beginners. Chaos (asymmetry of structure or color) is a useful concept that can be found in almost all algorithms, including algorithms for acral melanomas or nail matrix melanomas. Sometimes chaos goes under different names like, for example, asymmetry, irregular blotch, bizarre pattern, or atypical network. Like every other dermatoscopic clue, chaos is not 100% specific or sensitive for malignancy. A small but considerable number of nodular melanoma lack chaos. Chaos, however, is usually present in acral melanomas even if the classic melanoma clue for acral melanomas, the parallel ridge pattern, is missing. Nail matrix melanomas are also typified by chaotic arrangement of longitudinal pigmented lines of the nail plate. Chaos alone is usually not sufficient to diagnose pigmented malignant neoplasms. Important other clues are the Hutchinson sign or the micro-Hutchinson sign for nail matrix melanomas, angulated lines and prominent skin markings for in situ melanomas and gray circles for facial in situ melanomas. Flat facial lesions in particular may be difficult to diagnose. A new algorithm suggests, instead of focusing on clues to melanoma, to look for features of solar lentigo first and if they are absent to consider melanoma in situ. Dermatoscopy also improves the diagnosis of non-pigmented neoplasms and helps to distinguish different subtypes of basal cell carcinoma and poorly and well differentiated types of squamous cell carcinoma. Finally, dermatoscopic images can be used to train automated diagnostic systems that are able diagnose pigmented lesions without the need of human experts. Although prospective trials are currently sparse, it can be expected that approved automated diagnostic systems will soon be available to support human experts in the diagnosis of cutaneous neoplasms.
